# Health Care Providers’ Perspectives on Factors Influencing Diabetes Management Among Thai Pregnant Women

**DOI:** 10.1177/26884844251383424

**Published:** 2025-09-29

**Authors:** Jiraporn Lininger, Ratchanok Phonyiam, Sangthong Terathongkum

**Affiliations:** Ramathibodi School of Nursing, Faculty of Medicine Ramathibodi Hospital Mahidol University, Bangkok, Thailand.

**Keywords:** diabetes, management, multilevel, pregnant women, Thailand

## Abstract

**Background:**

Diabetes during pregnancy poses significant risks to maternal and fetal health. This study explores health care providers’ perspectives on factors influencing pregnant women with type 2 diabetes management in Thailand.

**Methods:**

A qualitative descriptive study based on the National Institute on Minority Health and Health Disparities (NIMHD) Research Framework. Semi-structured interviews were conducted with 13 physicians and nurses from two public hospitals (urban and rural areas). Directed content analysis was used to analyze the data, with themes and subthemes derived from initial codes based on the NIMHD framework.

**Results:**

Thirteen health care providers participated, including 10 nurses and 3 physicians. Three major themes emerged. Each theme was categorized into NIMHD domains of influence, including biological, behavioral, physical/built environment, and sociocultural environment. Theme 1: Individual-level factors include biological vulnerability and mechanisms in diabetes risk, maternal and neonatal complications, unplanned pregnancy and unawareness of preexisting diabetes, lifestyle behavior factors contributing to diabetes risk, diabetes management during pregnancy, environmental and workplace adjustments for pregnant women, sociodemographic challenges in diabetes management during pregnancy, and culture and beliefs in pregnancy care. Theme 2: Interpersonal-level factors include maternal responsibility for a safe pregnancy, family involvement in pregnancy care, promoting diabetes awareness and healthy lifestyle in schools and workplaces, and the role of social media and digital platforms in maternal health. Theme 3: Community-level factors include the role of community functioning in maternal health, access to healthy and safe food in the community, and cultural shifts toward convenience food options.

**Discussion:**

Emphasizing these factors requires a coordinated approach involving health care providers, families, communities, schools, workplaces, and policymakers. Tailored interventions promoting diabetes screening, healthy lifestyles, and supportive environments for pregnant women, particularly those from disadvantaged backgrounds, are crucial. Future research should focus on developing culturally sensitive, community-based strategies to overcome barriers to care and improve diabetes management during pregnancy.

## Introduction

Diabetes during pregnancy, especially preexisting type 2 diabetes mellitus (T2DM), presents significant risks to both maternal and fetal health.^1^ Pregnancies affected by T2DM have a higher rate of spontaneous abortion and perinatal mortality than those with type 1 diabetes mellitus.^2^ When compared to pregnancies with gestational diabetes mellitus (GDM), which is characterized by glucose intolerance recognized after 24 weeks of gestation, T2DM pregnancies are more likely to result in congenital anomalies and stillbirth.^3^ When T2DM goes undiagnosed or is poorly managed during pregnancy, it can result in preeclampsia and an elevated risk of neonatal morbidity.^1^ Preterm births (<37 weeks) occur four times more often in pregnant women with diabetes compared with those without this disease.^4^ Babies born to mothers with diabetes are more likely to be large for gestational age^5^ or macrosomia and experience metabolic issues, such as hypoglycemia.^6^ Given the global increase in diabetes, particularly among women of reproductive age, effectively managing T2DM during pregnancy to prevent complications has become a critical priority.

Despite national efforts to improve screening and care for pregnant women with diabetes, various factors—ranging from individual behaviors to community-level influences—can impact the effectiveness of these managements.^7,8^ The National Institute on Minority Health and Health Disparities (NIMHD) Research Framework is a comprehensive approach designed to address the complex and multifaceted nature of health, considering factors at the individual, interpersonal, and community levels across domains of influence, such as behavioral and physical/built environmental domains.^9^ The NIMHD framework has previously explored the experiences of pregnant women with T2DM in Thailand.^10^ Building on our previous study,^10^ it is crucial to gain a deeper understanding of health care providers’ perspectives on their experiences working with pregnant women with T2DM to address health challenges arising from individual factors, interpersonal factors involving family members and colleagues, and community factors involving neighborhood norms and lifestyle.

Health care providers, including physicians and nurses, play a critical role in managing pregnant women with T2DM and identifying challenges faced by women.^11,12^ Previous research from focus group discussions with T2DM patients highlighted the benefits of a multidisciplinary health care team and involving patients in self-care through a comprehensive diabetes education program.^11^ A meta-analysis of team-based care involving primary care providers, including physicians and nurses, in the management of diabetes showed a reduction in blood glucose levels, with a decrease of −0.5% in HbA1c (95% confidence interval = −0.7, −0.3).^13^ In Thailand, several factors influence the interaction between health care providers and patients, including cultural norms that stress respect and hierarchy, where patients often defer to their doctors’ decisions. Varying levels of access to health care based on socioeconomic status further impact the quality of care provided.^14^

Thus far, health care providers’ perspectives on the individual, interpersonal, and community factors affecting diabetes management during pregnancy remain underexplored in Thailand. This study aims to fill this gap by examining health care providers’ views on the factors influencing the care and outcomes of pregnant women with T2DM, highlighting factors for improving care at multiple levels.

### Research objectives

This study seeks to explore the perspectives of physicians and nurses on individual, interpersonal, and community factors related to managing diabetes in pregnant women.

## Materials and Methods

### Study design

This study employs Sandelowski’s (2000) qualitative description (QD) method, which offers a comprehensive summary by focusing on the “who, what, and where” of experiences.^15^ Rooted in naturalistic inquiry, this QD methodology is committed to studying phenomena in their natural context.^15^ This approach is utilized here to gain an in-depth and detailed understanding of Thai physicians’ and nurses’ perceptions of T2DM in pregnant women. The aim is to capture their perspectives while also identifying the factors they encounter in providing antenatal care.

The study protocol and materials received approval from the Institutional Review Board at Mahidol University (MURA.2024/102) before the study was carried out.

### Sample size and setting

Between May and December 2024, participants were selected using purposive sampling to ensure a diverse representation of experiences and backgrounds. This purposive sample included physicians and nurses who had provided care to pregnant women with T2DM at two public hospitals in Thailand. For the maximum variation sampling purpose, we recruited the participants from two different locations in the central part of Thailand. The first hospital was in a rural area and operated as a secondary hospital with inpatient units, but it had no intensive care unit or full-time Obstetrics and Gynecology physician. The second hospital was in an urban area and was considered a tertiary medical center. The recruitment process aimed to ensure a diverse sample, encompassing various health professions and participants from different hospitals and provinces.

The study aimed to interview 13 health care providers until data saturation was achieved. To participate, health care providers had to meet the following inclusion criteria: they must be physicians or nurses providing care to pregnant women with T2DM, be 18 years or older, and be able to speak Thai.

### Instruments

The study utilized two primary instruments. First, a demographic questionnaire was designed to gather information about participants’ sociodemographic and professional characteristics, including age, educational level, professional role, years of experience, and diabetes care training (specifically for T2DM in pregnancy). Second, the research team developed an interview guide (Table 1) based on the NIMHD framework’s levels of influence. The interview guide was pilot-tested with a registered nurse and revised as needed based on feedback.

**Table 1.** Interview Guide

Topics	Interview questions	Probing questions
Health care providers’ experiences	Tell me about your experience seeing pregnant women with T2DM.	Can you give me an example?
Individual-level factors	What are the common factors women face in managing their diabetes during pregnancy that are affected by their personal health behaviors?	Could you provide some examples and explain how they were addressed?
	What factors have you discovered that help to manage diabetes and improve health behaviors among pregnant women?	Could you provide some examples and describe their effectiveness?
Interpersonal-level factors	What are the common factors women face in managing their diabetes during pregnancy that are affected by their partner, family, peers, or coworkers?	Could you provide some examples and explain how they were addressed?
	What factors have you discovered that help to manage diabetes from their partner, family, peer, or coworkers?	Could you provide some examples and describe their effectiveness?
Community-level factors	What are the common barriers women face in managing their diabetes during pregnancy that are affected by their community?	Could you provide some examples and explain how they were addressed?
	What factors have you discovered that help in managing diabetes in their community?	Could you provide some examples and describe their effectiveness?
Additional thoughts	Is there anything else you want to share, or do you have any other questions?	None

T2DM, type 2 diabetes mellitus.

### Data collection

Before participants decided to participate, the research team explained the study’s objectives, data collection procedures, potential benefits, and overall impact. We highlighted participants’ rights to participate or withdraw from the study at any time without impacting their careers. Participants were also allowed to ask questions before joining the study.

Semi-structured interviews were conducted in person at the antenatal clinic. Two interviewers met with each participant in a private room. One interviewer served as the main interviewer, while the other assisted with probing questions and observation. The two interviewers were native Thai speakers with nursing backgrounds, doctoral degrees, and experience in interactive data collection and report writing. Each interview lasted up to 60 minutes and was conducted in Thai to ensure participants could express themselves comfortably and naturally. The interviewers employed active listening techniques and probing questions to encourage participants to share their experiences in depth. All interviews were audio-recorded with the participants’ consent to ensure comprehensive data capture. For participants unable to attend in person, phone interviews were recorded using Voice Memo after obtaining their permission. This interview approach was chosen to allow participants the freedom to describe, explain, and share their perspectives and experiences. Participants were encouraged to elaborate on their experiences in an open-ended manner, resulting in detailed narratives. Interview length varied based on the extent of participants’ sharing. Field notes documenting the tone and atmosphere of each conversation were taken immediately after each interview.

### Data management

A research assistant transcribed the audio recordings verbatim in Thai. All nonverbal cues, such as pauses and laughter, were noted in each transcript. A bilingual researcher cross-checked the translated transcripts against the original recordings to ensure fidelity and accuracy, thereby verifying the authenticity and clarity of the participants’ narratives. All identifiable information, including participants’ names and hospital settings, was removed, and each interview file and questionnaire was labeled sequentially with a three-digit identification number (*e.g.*, ID001). Participants were tracked using these case identification numbers. Interview data (audio files) and transcribed interviews were stored on a secure server. We provided assurances of individual participant privacy and confidentiality, and data results are reported only in aggregate form.

### Data analysis

For quantitative data, information from the demographic questionnaire was entered and analyzed using IBM SPSS version 25.0. The analyses included calculating means, standard deviations (SDs), and ranges for continuous variables and frequencies and percentages for categorical variables.

Interview transcripts were analyzed using Atlas.ti version 9, a qualitative data analysis software that facilitated data organization and collaborative text analysis. Data analysis was based on directed content analysis, an approach where analysis begins with a theory or relevant research findings to guide initial codes.^16^ In this study, we utilized the NIMHD framework’s levels of influence—individual, interpersonal, and community—as our initial coding framework. Factors were defined as elements that either impede pregnant women in managing their diabetes, pose challenges for health care providers in delivering care, or support and encourage women in managing their diabetes and assist health care providers in their care delivery.

One coder independently coded the interview transcripts, while another coder is the artificial intelligence (AI)-generated feature from Atlas.ti. Before we analyzed all 13 interview transcripts, we conducted a pilot test with three interview transcripts (ID001, ID002, and ID003). We used the “*Conversational AI*” feature. We set the prompt as, “*Please analyze the data into factors influencing diabetes during pregnancy at the individual, interpersonal, and community levels based on the NIMHD framework.*” The results revealed some initial patterns, such as “*lack of awareness and education*” and “*financial constraints*,” which were categorized under “*individual-level factors*.”

Following pilot testing, we analyzed all interview transcripts and provided a summary to a third coder, a nursing researcher who reviewed the codes and helped resolve discrepancies. We identified recurring patterns in participants’ responses based on their relevance to the predetermined domains of inquiry. Descriptive coding was employed to assign a single code to each data segment (two to three lines) that conveyed a complete, stand-alone meaning. The analysis involved a continuous iterative process of moving between the entire dataset and individual transcript coding. The coders systematically worked through the dataset, giving equal attention to each transcript and identifying salient aspects that could form the basis of recurring patterns across the dataset. They then developed clear definitions and names for each theme by identifying its essence and determining the aspects that captured health care providers’ perspectives. The themes were reviewed and refined through this iterative process to ensure they accurately represented the data and were both interrelated and distinct. Researchers engaged in discussions and peer debriefing sessions to validate the themes and resolve any differing opinions about the data that informed the themes. Finally, the themes were defined and named, providing a comprehensive understanding. Each theme was supported by direct participant quotes to illustrate key points and ensure the findings were data-driven. The final themes were then integrated into a cohesive narrative that captured the complex nature of health care providers’ experiences. A table presenting the themes, subthemes, and supporting quotations was developed.

This study did not involve member checking, as the findings were drawn from multiple participants to identify common themes, not individual accounts. Practical limitations made recontacting participants difficult.^17^ Furthermore, the impact of member checking on a study’s credibility and findings is often limited and inconsistent, with participants potentially changing their views or feeling pressured by the researcher’s interpretations.^18^

### Rigor

The rigor of this analysis was strengthened through several steps. First, the research team collaboratively reviewed the data coding, leading to iteratively refined and consensus-driven final themes. To further demonstrate rigor and trustworthiness, the presented themes and subthemes were linked to supporting data extracts. Second, after the analysis and coding team agreement, key quotations were translated from Thai to English by two qualitative PhD holders. Last, the second author compared and adjusted these translations to ensure accuracy before the article’s completion.

## Results

### Sample characteristics

A total of 13 health care providers participated in this study: 7 were from a secondary hospital in a rural area, while 6 worked at a tertiary medical center located in an urban area. Of those, 10 were nurses (8 registered nurses and 2 advanced practice nurses), and 3 were physicians (a medicine, an obstetrician, and an endocrinologist). Their educational attainments were as follows: bachelor’s degree (*N* = 8; 61.5%), master’s degree (*N* = 2; 15.4%), doctoral degree (*N* = 2; 15.4%), and certification in endocrine expertise (*N* = 1; 7.7%). Most participants were female (*N* = 11; 84.6%). Their average age was 46.08 years (SD = 12.42), ranging from 24 to 63 years. They had worked in their profession and cared for pregnant women with diabetes for an average of 12.92 years (SD = 9.99), with a range from 1 to 32 years. More than half of the participants (*N* = 9; 69.2%) reported no previous training experience regarding type 2 diabetes in pregnancy.

### Qualitative findings

**FIG. 1.**
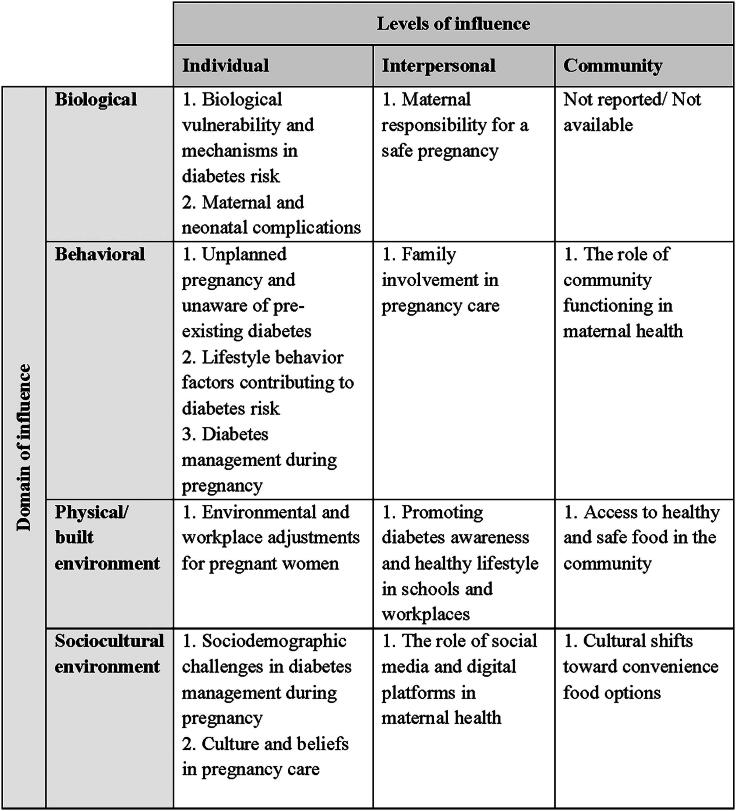
Qualitative findings.

Three major themes have emerged from 13 participants: individual-level, interpersonal-level, and community-level factors. Each level was categorized into four different domains of influence, including biological, behavioral, physical/building environment, and sociocultural environment, according to the NIMHD framework (Fig. 1). The quotations of participants from two hospitals, along with the summative interpretation, are described in Table 2.

**Table 2.** Quotations and Summative Interpretation

Themes/subthemes	Quotations from health care providers		Summative interpretation
	Rural area, secondary hospital (ID001–ID007)	Urban area, tertiary hospital(ID008–ID013)	
Theme 1: Individual-level factors			
• Biological vulnerability and mechanisms in diabetes risk	“We need to check for genetic factors, a family history of diabetes, and whether the person is overweight” (ID003). “Managing blood sugar in pregnant women with type 2 diabetes... may lead to issues with blood sugar dropping because pregnant women release insulin differently” (ID004).	“These individuals likely have a BMI of 30 or more and are probably over 40 years old” (ID008). “The first three months of pregnancy are usually the most difficult for controlling blood sugar because the hormones during pregnancy are also a factor” (ID008).	Participants from both hospitals mentioned several indicators, including genetics, family history, and the risk of blood sugar drops in pregnant women. The focus was also on BMI, age, and hormonal changes.
• Maternal and neonatal complications	“The abnormal symptoms we encountered are dizziness, palpitations, or sudden sweating on the face” (ID001).	“This results in a larger incision and a longer hospital stay” (ID009). “Sometimes the pregnancy ends in miscarriage, or sometimes, during the pregnancy, we find out the embryo is no longer there, and then the woman has to face a miscarriage” (ID011). “The baby will definitely have hypoglycemia, no doubt about it, because there is still a lot of insulin during delivery. If blood sugar is not well managed during pregnancy, there will be problems” (ID013).	Secondary hospital participants concentrated on the immediate symptoms of hypoglycemia. In contrast, tertiary hospital participants focused on broader outcomes and complications related to pregnancy and delivery, such as miscarriages, the need for larger incisions, and hypoglycemia in newborns.
• Unplanned pregnancy and unaware of preexisting diabetes	“Most of the time, we find out that when they already had diabetes before pregnancy and are just coming for prenatal care. This is what we encounter” (ID001).	“Some never had health checks and discover their HbA1c is high... they did not know they had diabetes before pregnancy” (ID009). “For unplanned pregnancies, managing blood sugar may not be as effective” (ID012).	A key similarity is that both rural and urban hospitals encounter pregnant women who are unaware they have diabetes before coming in for care.
• Lifestyle behavior factors contributing to diabetes risk	“The trend of increasing diabetes is likely due to diet and eating habits from adolescence. Teenagers tend to like sugary drinks, which can trigger diabetes” (ID001). “Most of them like sweetened beverages. They say they feel tired, and if they do not drink it, they lack energy. I usually suggest reducing intake or drinking more water” (ID003).	“Children who eat fast food—do they have any activity at home or in their condo? Many condos even have their own fitness center” (ID013).	Providers from both hospitals recognize that lifestyle factors (such as eating habits and physical activity) contribute to diabetes.
• Diabetes management during pregnancy	“Control the diet, we will order food from the hospital’s kitchen” (ID001).	“We ask patients to take pictures of their food and measure rice portions. They must follow a meal plan with 2 servings of vegetables, 1 serving of carbohydrates, and 1 serving of protein” (ID011). “For the T2DM group, we focus on strict self-care, teaching them to follow nutritional guidelines, monitor blood sugar, and inject insulin if needed” (ID008).	Both settings emphasize diet control to manage diabetes. However, the methods of implementation differ significantly. The secondary hospital takes a more direct approach by ordering food from the hospital kitchen for women. In contrast, the tertiary hospital focuses on patient-led self-care, nutritional guidelines, and blood sugar monitoring.
• Environmental and workplace adjustments for pregnant women	“It depends on the environment around the pregnant woman. Advertisements, social media, and reviews, like finding out where to get a hot pot, have an impact” (ID001). “We should arrange time for them to rest. Standing for long periods causes swelling and poor circulation. Pregnant women may need job adjustments, such as moving from the production line to a QC [Quality Control] position and be given time to walk around” (ID004).	“Patients cannot return to contact us due to work obligations. They may come once, then disappear, and only show up again when giving birth” (ID009).	Both hospitals share the challenge of environmental factors impacting a pregnant woman’s health, such as media and social environments, and a woman’s work environment, including the physical demands of her job, which can lead to health issues. The participants from the tertiary hospital mentioned work-related issues contributing to a lack of follow-up care.
• Sociodemographic challenges in diabetes management during pregnancy	“Sometimes they do not attend their appointments because if they take time off work, they lose 300 to 400 Baht. If they run out of medication, they can come to get more, but we do not want them to miss insulin injections” (ID003). “If they lose income, we offer alternatives like evening clinic visits after work” (ID007).	“Most patients have housing problems because sometimes they have frequent appointments and no place to stay in Bangkok. Patients from other provinces must rent hotels or similar accommodations” (ID008). “Patients from other provinces usually do not stay overnight unless they have relatives. They often come early, finish appointments, and leave the same day” (ID010).	Both hospitals face challenges with patient attendance, but the reasons differ significantly. The rural hospital addresses the issue of lost income due to missed work by offering evening clinics. In contrast, the urban hospital grapples with the logistical and financial burden of travel and accommodation costs for patients coming from other provinces, which impacts their ability to attend frequent appointments.
• Culture and beliefs in pregnancy care	“Ginger is recommended during the first three months to prevent vomiting, and drinking ginger water refreshes them. Meals typically include plain rice and fish, and avoid coconut milk” (ID004). “In the Thai context, sweet drinks are widely available” (ID007).	“If they can understand why something happens a certain way, and why their previous understanding was incorrect—not just for the sake of belief, but understanding why it’s not right—that would be great. However, it takes time to counsel them. For example, with pregnant women, there might be foods they can or cannot eat” (ID013).	Both hospitals shared about cultural and belief systems related to pregnancy care, such as what to eat and what food to avoid, based on the Thai context.
Theme 2: Interpersonal-level factors			
• Maternal responsibility for a safe pregnancy	“A woman preparing for pregnancy will prioritize her child and make an effort to manage her diabetes independently” (ID005).	“The patient will ask if the injection affects the baby or if it is dangerous. We advise them to follow all necessary steps, and they are happy to do so because they want a healthy baby” (ID011).	Participants from both hospitals recognize that a woman’s motivation to manage her diabetes during pregnancy is her desire to have a healthy baby.
• Family involvement in pregnancy care	“When I advise the patient to take care of themselves at home if they have a family with them, I will tell them, ‘This is how it is, you need to pay attention to this too,’, especially with food, since they eat together” (ID001). “We will ask, ‘How does your family eat?’ We encourage them to prepare meals together. If the patient has diabetes, the whole family, even children, may be at higher risk” (ID007).	“If the father is there, he might say, ‘Hey, can you avoid sugary drinks for a bit?’ He helps monitor the mother’s diet and administer insulin” (ID012).	Participants from both hospitals recognize that family involvement is crucial for a pregnant woman’s health, such as with eating habits, meal preparation, and insulin injection.
• Diabetes awareness and healthy lifestyle in schools and workplaces	“Regarding diabetes in children, we need school health programs. Teachers should guide students on how much rice, protein, and vegetables to eat” (ID004). “School health will help with education, and a social medicine group at the hospital handles this” (ID006).	“A patient works until 10:00 PM, and the company provides free meals at the cafeteria. They eat dinner at 6:00 PM and then have to return for a blood draw at 10:00 PM. They complained, ‘I finished eating and had to walk.’ It was difficult because they still had to work” (ID009).	Participants from two hospitals acknowledged the importance of promoting healthy lifestyles outside of the clinical setting.
• The role of social media and digital platforms in maternal health	“They watch TikTok and follow it. People share their experiences, which can influence how mothers care for themselves” (ID002). “Platforms like Instagram and TikTok promote supplements. We recommend patients bring the supplement label to check the ingredients if unsure” (ID003).	“We offer the opportunity for patients to ask health-related questions *via* the Line app, helping them act on information promptly” (ID011).	Both hospitals recognize social media’s influence on maternal health, but their engagement differs. Rural hospitals view platforms like TikTok as a source of misinformation they must counteract. In contrast, urban hospitals proactively use digital platforms to provide patients with direct access to professional medical advice and support.
Theme 3: Community-level factors			
• The role of community functioning in maternal health	“The community must support, encourage, and help care for pregnant women” (ID003). “When a child is born, the community must be ready; it should be a good community” (ID004).	“Pregnant women from other provinces come for prenatal care, then after giving birth, they return to Bangkok to work. They might be seven or eight months pregnant and go back to their hometown to give birth, or they might give birth at our hospital. After that, they work in Bangkok as a single family, taking three months of maternity leave. They go back to stay in their home province with the grandparents. Then they return to work while the grandchild stays in the province. This is different from those in the provinces who live in their local areas. The Village Health Volunteers definitely have an impact. So, we have these kinds of patient groups that are quite different” (ID008).	The role of community support in maternal health differs between rural and urban areas. Rural hospitals rely on a cohesive, local community to support pregnant women. Conversely, urban hospitals deal with the challenges of a fragmented community, as migrant workers are often separated from their families and support networks.
• Access to healthy and safe food in the community	“The community must have restaurants that serve pesticide-free vegetables. There should be more vegetable shops because vegetables are expensive and unsafe. Few people can grow vegetables; it is rare” (ID004).	“In the city, it’s about meals like greasy grilled pork and sticky rice, or curry dishes where you can’t ask for a non-creamy, coconut-free option. You won’t easily find a dry, oil-free stir-fried basil dish” (ID009).	Both rural and urban areas struggle with access to healthy food, but the specific challenges differ. Rural areas face a lack of affordable, safe vegetables, while urban areas have difficulty finding healthy, low-fat foods.
• Cultural shifts toward convenience food options	“With Thai Food Delivery Markers, ordering food is easy. Even a convenience store encourages unhealthy eating, potentially leading to diseases” (ID002). “There are two or three convenience stores in the community, but we still lack healthy food options. Our farm grows pesticide-free vegetables, which are sold in department stores” (ID004).	“Regarding the change in food, Thai people are now eating more fast foods and delivery foods” (ID013).	Both mentioned a significant cultural shift toward convenience food options.

BMI, body mass index.

Theme 1: Individual-level factors had four domains as follows:

1.1 The biological domain had two subthemes. The first is biological vulnerability and mechanisms in diabetes risk, focusing on biological factors that increase diabetes risk. Participants from both hospitals mentioned several indicators, such as family history, age, and overweight/obesity, highlighting the need for diabetes screening. Health care providers noted, “*We need to check for genetic factors, a family history of diabetes, and whether the person is overweight*” (ID003) as well as “*a BMI of 30 or more and age older than 40 years old*” (ID008). Managing blood sugar in pregnant women with preexisting T2DM is particularly challenging due to hormonal changes during pregnancy, which affect insulin release and resistance and may cause fluctuations in blood sugar. As one participant explained, “*Managing blood sugar in pregnant women with type 2 diabetes… may lead to issues with blood sugar dropping because pregnant women release insulin differently*” (ID004).

The second subtheme is maternal and neonatal complications. Pregnant women with T2DM face significant problems with both their health and that of their babies. Secondary hospital’s participant concentrated on the immediate symptoms of hypoglycemia, describing symptoms as, “*The abnormal symptoms we encountered are dizziness, palpitations, or sudden sweating on the face*” (ID001). In contrast, participants from a tertiary hospital focused on broader outcomes and complications related to pregnancy and delivery, with one participant explaining, “*This results in a larger incision and a longer hospital stay*” (ID009). Neonatal complications, such as hypoglycemia, hypoxia, birth injuries, and macrosomia, are common when blood sugar is poorly managed. As one participant noted, “*The baby will have hypoglycemia, no doubt about it, because the insulin level remains high during delivery. If blood sugar is not well managed during pregnancy, there will be problems*” (ID013).

1.2 The behavioral domain had three subthemes. The first is an unplanned pregnancy and unawareness of preexisting diabetes. A key similarity is that both rural and urban hospitals encounter pregnant women who are unaware they have T2DM before coming in for care, as high blood sugar is often discovered during the first trimester. This highlights unplanned pregnancies and those where women have not monitored their health or prepared for blood sugar control. As a result, some women may be unaware of preexisting diabetes, making blood sugar management during pregnancy more challenging. One health care provider shared, “*For unplanned pregnancies, managing blood sugar may not be as effective*” (ID012), while another noted, “*Some never had health checks and discover their HbA1c is high… they did not know they had diabetes before pregnancy*” (ID009).

The second subtheme is lifestyle behavior factors contributing to diabetes risk. Health care providers from both hospitals recognize that lifestyle behavior factors (such as an unhealthy diet and less physical activity) contribute to diabetes. Unhealthy diets, including frequent consumption of sugary drinks, fast food, and sweets, along with sedentary lifestyles—particularly among adolescents and factory workers—significantly increase the risk of diabetes. One participant noted, “*The trend of increasing diabetes is likely due to diet and eating habits from adolescence. Teenagers tend to like sugary drinks, which can trigger diabetes*” (ID001). Others reported that adolescents often feel tired and lack energy, with one participant explaining, “*Most of them like sweetened beverages. They say they feel tired, and if they do not drink it, they lack energy. I usually suggest reducing intake or drinking more water*” (ID003). In urban areas, the lack of exercise opportunities is also a concern; as one participant pointed out, “*Children who eat fast food—do they have any activity at home or in their condo? Many condos even have their own fitness center*” (ID013).

The third subtheme in the behavioral domain is diabetes management during pregnancy. Both settings emphasize diet control to manage diabetes. However, the methods of implementation differ significantly. The secondary hospital takes a more direct approach by ordering food from the hospital kitchen for women. One shared, “*Control the diet, we will order food from the hospital’s kitchen*” (ID001). In contrast, the tertiary hospital focuses on patient-led self-care, nutritional guidelines, and blood sugar monitoring. Pregnant women are taught to follow a balanced meal plan and monitor their blood sugar, with health care providers’ support to ensure their health and the baby’s safety. One participant explained, “*For the T2DM group, we focus on strict self-care, teaching them to follow nutritional guidelines, monitor blood sugar, and inject insulin if needed*” (ID008). Another added, “*We ask patients to take pictures of their food and measure rice portions. They must follow a meal plan with 2 servings of vegetables, 1 serving of carbohydrates, and 1 serving of protein*” (ID011).

1.3 The physical/built environment domain identifies environmental and workplace adjustments for pregnant women as a key subtheme. Participants from both hospitals shared that environmental factors, such as social media and advertisements, challenge pregnant women’s health and influence their behaviors. One participant noted, “*It depends on the environment around the pregnant woman. Advertisements, social media, and reviews, like finding out where to get a hot pot, have an impact*” (ID001). The subtheme also emphasizes the importance of workplace accommodations, especially for jobs requiring long-standing periods. One participant explained, “*We should arrange time for them to rest. Standing for long periods causes swelling and poor circulation. Pregnant women may need job adjustments, such as moving from the production line to a QC [Quality Control] position, and be given time to walk around*” (ID004). The participant from the tertiary hospital mentioned work-related issues contributing to a lack of follow-up care: “*Patients cannot return to contact us [health care providers] due to work obligations*” (ID009).
1.4 The sociocultural environment domain has two subthemes. The first identifies sociodemographic challenges in diabetes management during pregnancy as a key subtheme. Both hospitals face challenges with patient attendance, but the reasons differ significantly. The rural hospital addresses issues such as economic constraints and employment-related income loss. Two participants noted, “*Sometimes they do not attend their appointments because if they take time off work, they lose 300 to 400 Baht. If they run out of medication, they can come to get more, but we do not want them to miss insulin injections*” (ID003). These barriers are more pronounced for pregnant women from rural or lower-income backgrounds. One participant shared, “*If they lose income, we offer alternatives like evening clinic visits after work*” (ID007). In contrast, the urban hospital grapples with the logistical and financial burden of travel and accommodation costs for patients coming from other provinces to the city, which impacts their ability to attend frequent appointments. For pregnant women traveling from rural areas to Bangkok, staying overnight can be challenging: “*Patients from other provinces usually do not stay overnight unless they have relatives. They often come early, finish appointments, and leave the same day*” (ID010). One participant supported, “*Most patients have housing problems because sometimes they have frequent appointments and no place to stay in Bangkok. Patients from other provinces must rent hotels or similar accommodations*” (ID008).

Second, the culture and beliefs in the pregnancy care subtheme reflect how cultural beliefs, family dynamics, and traditional practices influence pregnancy care, as mentioned by participants from both hospitals. In Thai culture, extended families often influence meal preparation, such as cooking in large quantities, like making a big pot of curry and dessert. One participant explained, “*In big families, meals are often prepared in large quantities*” (ID004). Additionally, cultural practices guide food choices to manage weight and symptoms during pregnancy. For example, women may eat salted vegetables, banana blossom, or ginger water to avoid excessive weight gain in the first trimester. These foods are believed to help with milk production and prevent vomiting. One participant shared, “*Ginger is recommended during the first three months to prevent vomiting, and drinking ginger water refreshes them. Meals typically include plain rice and fish, and avoid coconut milk*” (ID004). These cultural practices shape pregnant women’s experiences and health care decisions.

Theme 2: Interpersonal-level factors have four domains as follows:

2.1 The biological domain includes the subtheme of maternal responsibility for a safe pregnancy, which highlights the mother’s crucial role in managing her health for the safety of both herself and her fetus. One participant explained, “*A woman preparing for pregnancy will prioritize her child and make an effort to manage her diabetes independently*” (ID005). Another added, “*The patient will ask if the injection affects the baby or if it is dangerous. We advise them to follow all necessary steps, and they are happy to do so because they want a healthy baby*” (ID011). Participants from both hospitals emphasize the mother’s proactive role in following medical guidance and managing diabetic conditions.
2.2 The behavioral domain emphasizes family involvement in pregnancy care. Participants from both hospitals recognize that family involvement is crucial for a pregnant woman’s health, especially husbands, in supporting the mother’s health. One participant shared, “*When I advise the patient to take care of themselves at home if they have a family with them, I will tell them, ‘This is how it is, you need to pay attention to this too,’ especially with food, since they eat together*” (ID001). This subtheme focuses on collaborative care, where family members assist with diet, meal preparation, and emotional support. One participant noted, “*We will ask, ‘How does your family eat?’ We encourage them to prepare meals together. If the patient has diabetes, the whole family, even children, may be at higher risk*” (ID007). Additionally, one participant mentioned, “*If the father is there, he might say, ‘Hey, can you avoid sugary drinks for a bit?’ He helps monitor the mother’s diet and administer insulin*” (ID012).
2.3 The physical/built environment domain promotes diabetes awareness and healthy lifestyles in schools and workplaces. Participants from two hospitals acknowledged the importance of promoting healthy lifestyles outside of the clinical setting. Education and support systems are key in managing and preventing diabetes in these environments. One participant shared, “*Regarding diabetes in children, we need school health programs. Teachers should guide students on how much rice, protein, and vegetables to eat*” (ID004). Another added, “*School health will help with education, and a social medicine group at the hospital handles this*” *(ID006).* Workplace support is also critical. One participant described a challenging situation: “*A patient works until 10:00 PM, and the company provides free meals at the cafeteria. They eat dinner at 6:00 PM and then have to return for a blood draw at 10:00 PM. They complained, ‘I finished eating and had to walk.’ It was difficult because they still had to work*” (ID009).
2.4 The sociocultural environment domain highlights the role of social media and digital platforms in maternal health. Both hospitals recognize social media’s influence on maternal health, but their engagement differs. Rural hospitals view platforms as a source of health-related information. Social media influences, such as Line app, TikTok, and Facebook, allow mothers to share experiences and health advice, which can be beneficial and harmful. One participant noted, “*They watch TikTok and follow it. People share their experiences, which can influence how mothers care for themselves*” (ID002). Another mentioned, “*Platforms like Instagram and TikTok promote supplements. We recommend patients bring the supplement label to check the ingredients if unsure*” (ID003). In contrast, urban hospitals proactively use digital platforms to provide patients with direct access to professional medical advice and support. Apps such as “Line” also provide accessible channels for patients to ask health-related questions and receive timely, reliable information. One participant shared, “*We offer the opportunity for patients to ask health-related questions via the Line app, helping them act on information promptly*” (ID011).

Theme 3: Community-level factors have three domains since the biological domain is unavailable.

3.1 The behavioral domain identifies the role of community functioning in maternal health, emphasizing the importance of community support for pregnant women and their families. The role of community support in maternal health differs between rural and urban areas. The rural hospital relies on a cohesive, local community to support pregnant women. One participant shared, “*The community must support, encourage, and help care for pregnant women*” (ID003). Another noted, “*When a child is born, the community must be ready; it should be a good community*” (ID004). Conversely, the urban hospital deals with the challenges of a fragmented community, as migrant workers are often separated from their families and support networks. One shared, “*Pregnant women from other provinces come for prenatal care, then after giving birth, they return to Bangkok to work…This is different from those in the provinces who live in their local areas. The Village Health Volunteers definitely have an impact. So, we have these kinds of patient groups that are quite different*” (ID008).
3.2 The physical/built environment domain identifies access to healthy and safe food in the community as a key subtheme. Both rural and urban areas struggle with access to healthy food, but the specific challenges differ. Rural areas face a lack of affordable and safe vegetables, one participant shared, “*The community must have restaurants that serve pesticide-free vegetables. There should be more vegetable shops because vegetables are expensive and unsafe. Few people can grow vegetables; it is rare*” (ID004). Urban areas have difficulty finding healthy, low-fat foods: “*In the city, it’s about meals like greasy grilled pork and sticky rice, or curry dishes where you can’t ask for a non-creamy, coconut-free option. You won’t easily find a dry, oil-free stir-fried basil dish*” (ID009).
3.3 The sociocultural environment domain identifies cultural shifts toward convenience food options as a key subtheme. Participants from both hospitals mentioned a significant cultural shift toward convenience food options. This highlights the contrast between the convenience of modern food delivery services and the need for healthier, sustainable food choices. One participant noted, “*With Thai Food Delivery Markers, ordering food is easy. Even a convenience store encourages unhealthy eating, potentially leading to diseases*” (ID002). Another shared, “*There are two or three convenience stores in the community, but we still lack healthy food options. Our farm grows pesticide-free vegetables, which are sold in department stores*” (ID004).

## Discussion

This study provides valuable insights into the multifactorial nature of diabetes management during pregnancy, highlighting key factors based on the NIMHD framework at the individual, interpersonal, and community levels. These factors, spanning from biological vulnerabilities to cultural influences and social determinants, shape maternal health outcomes, particularly for pregnant women with diabetes.

At the individual level, the study revealed the biological vulnerabilities that increase the risk of diabetes, including family history, age, and overweight/obesity. This aligns with global research^19^ and Thailand^20^ that recognizes these factors as significant risk determinants for T2DM. The challenges in managing blood sugar during pregnancy, particularly during the first trimester, are well-documented. The hormonal changes during pregnancy complicate glycemic control,^21^ and this study’s findings reflect the difficulties in managing blood sugar, especially among women with preexisting diabetes. Research has similarly pointed to increased insulin resistance during pregnancy, which makes blood sugar management more difficult.^22^ The need for early screening, particularly in women with risk factors, becomes crucial to improving diabetes management.^23^ Health care providers should monitor risk factors for diabetes and support blood sugar management, especially during pregnancy, to address challenges resulting from hormonal changes and insulin resistance.

The study highlighted unplanned pregnancies and unawareness of preexisting T2DM as key issues. Unplanned pregnancies are often linked to impaired preconception health and inadequate diabetes management. A previous scoping review found that only 32% of women with pregestational type 1 diabetes attended prepregnancy care.^24^ Barriers were identified, including patient factors such as lack of knowledge, negative perceptions of health care, and unclear attendance pathways.^24^ According to the American Diabetes Association, pregnant women are screened for GDM at 24 weeks of gestation (the second trimester).^25^ If high blood sugar levels are detected before 24 weeks, the condition is considered preexisting diabetes.^25^ Consistent with global trends, where about half of women are diagnosed with T2DM during pregnancy in any trimester,^26^ many participants in this study said that pregnant women were also unaware of their diabetes until they became pregnant. This lack of prior knowledge made it challenging to manage blood sugar levels effectively. Preexisting diabetes first detected during pregnancy was strongly linked to a higher risk of fetal overgrowth.^26^ It emphasizes the importance of early and comprehensive diabetes screening, even in women without a clear diagnosis before pregnancy.^27^ Policy implementation should focus on improving access to prepregnancy care and diabetes screening and addressing barriers such as lack of knowledge and negative perceptions of health care.

Health care providers acknowledged that sociodemographic challenges among pregnant women constitute a key barrier to accessing care. Although Thailand offers universal health coverage, pregnant women still contend with the financial burden of indirect costs.^28^ Our study revealed that women attending clinic appointments frequently must take leave from work, consequently impacting their daily income. For women residing outside the city who require hospital care, the expenses for accommodation and travel, along with the need to manage their time for a full day or two, present a significant challenge. This finding is similar to a previous study in that Thai adults with diabetes in middle adulthood—a life stage characterized by multiple responsibilities—also faced similar challenges.^29^ Collaborating with pregnant women to arrange a convenient time for their antenatal clinics will be a beneficial practice.

The challenges in diabetes management during pregnancy, such as dietary control, blood sugar monitoring, and insulin administration, were discussed by the participants who are health care providers. The proactive role of health care providers in guiding women to manage their diabetes through diet and self-care aligns with global guidelines for pregnancy care in women with diabetes.^1^ However, these efforts are often complicated by pregnancy’s physical and emotional burdens, underscoring the importance of continuous support from health care providers and family members to ensure adherence to diabetes management protocols.^12^ In the Thai context, where extended families play a significant role, health care providers should offer continuous guidance on diabetes management during pregnancy, addressing physical and emotional challenges while collaborating with family members to ensure adherence to care protocols and provide a supportive environment.

One of the interesting findings is that 69.2% of our participants reported no previous specific training in managing T2DM during pregnancy. This is similar to a previous study in Morocco, where only 36.1% of health care providers received relevant training during their studies, and only 10.2% received on-the-job training for GDM.^30^ Similarly, a qualitative study in a central hospital in Zimbabwe reported a significant lack of personnel trained in managing diabetes in pregnancy, noting that some specialists, such as endocrinologists, podiatrists, and diabetes educators, were unavailable in that setting.^31^ Thus, to ensure that pregnant women with T2DM receive proper care, the available and comprehensive training of health care providers needs to be initiated or scaled up.

The study highlighted the critical role of the family in supporting diabetes management during pregnancy, aligning with previous reviews showing the importance of care and affection from husbands and family.^32^ Health care providers should tailor social support interventions to meet individualized needs, as these can vary. The involvement of partners, particularly husbands, in dietary adjustments, emotional support, and active participation in care is crucial. Participants reported that family engagement in meal planning and healthy eating improved health outcomes, emphasizing the positive impact of family support on managing diabetes. A previous meta-synthesis found that enhancing family members’ knowledge alone may not support people with T2DM. Therefore, family interventions should extend beyond just educating family members to also focus on improving family dynamics and supportive behaviors.^33^ The health care provider’s role is to design and implement individualized social support interventions that engage family members, particularly partners, in managing diabetes while also promoting family-centered care to enhance health outcomes for pregnant women with diabetes.

At the community level, the findings emphasize the role of the physical and built environment, access to health care, and sociocultural influences on maternal health. The access to healthy and safe food in the community subtheme underlines the importance of community-based initiatives to provide affordable, nutritious food options.^34^ The food environment, including food insecurity and limited access to healthy food, is crucial to explore, as the lack of availability of safe, pesticide-free vegetables and the high cost of nutritious foods create significant obstacles to healthy eating.^35^ Community-driven interventions to increase access to healthy, affordable food—such as farmers’ markets, food cooperatives, or subsidies for nutritious food^36^—could help address these barriers and improve maternal health outcomes. Health care providers should support and work with community-driven initiatives to improve access to healthy food and address barriers, ultimately enhancing maternal health outcomes.

This study emphasizes the sociocultural environment and the influence of modern convenience food options on dietary choices. The popularity of food delivery services, such as Grab and Line Man, particularly since the COVID-19 pandemic and climate change, has made these services the preferred option for many restaurant customers in Thailand.^37^ While food delivery to people’s doorsteps offers convenience, it has contributed to unhealthy eating habits, particularly due to the dominance of fast-food options.^38^ The impact of convenience foods on health is an increasing global concern, as processed foods high in sugar, fat, and salt are contributing to rising obesity rates, which are a significant risk factor for diabetes.^39^ Collaboration across disciplines and sectors is crucial in developing comprehensive solutions to improve maternal health.^39^ The shift toward more sustainable and healthy food choices within communities, through initiatives such as local farms or healthier food delivery options, could help mitigate the impact of these convenience food trends.^40^ Health care providers should collaborate across disciplines and support initiatives promoting sustainable, healthy food choices to mitigate the risk of unhealthy eating habits and improve overall health.

The role of community functioning in supporting maternal health underscores the importance of a supportive environment. This includes the role of the community in encouraging healthy practices and providing a network of support for pregnant women.^41^ Previous studies have demonstrated that robust social networks and community support systems greatly enhance maternal outcomes, such as subjective well-being and mental health protection.^5^ Our study also highlights a difference in the role of community support for maternal health in rural versus urban areas. This finding is important for policymakers and health care providers to consider when designing community-based programs.^42^ For those in rural areas, telehealth is a potential complementary tool that can minimize gaps in quality of care, which disproportionately impact rural-dwelling women.^42^ Therefore, creating supportive communities where pregnant women can access education, health care, and social support is vital for improving maternal health outcomes, particularly for those with diabetes.

### Strengths and limitations

This study provides a comprehensive, multilevel perspective on diabetes management during pregnancy, considering individual, interpersonal, and community factors. Using real-world insights from health care providers offers practical, clinically relevant findings. Focusing on the Thai context adds cultural specificity, making the results valuable for local health care practices. The study highlights actionable areas for intervention, such as early diabetes screening, family involvement in care, efforts within schools and workplaces, and community-based interventions. These strategies aim to enhance awareness, improve care management, and address barriers to diabetes control, ultimately promoting healthier outcomes for pregnant women with diabetes.

Some limitations need to be noted. First, the findings are context-specific to Thailand and may not directly apply to other regions with different health care systems or cultural practices. Second, the cross-sectional design prevents long-term insights, and a more detailed exploration of sociodemographic factors could have provided a richer understanding. Last, providers may emphasize expected good practices, which may result in reporting bias.

## Conclusions

This study highlights the complex nature of diabetes management during pregnancy, where multiple factors significantly influence the health outcomes of pregnant women with T2DM. Addressing these factors requires a coordinated approach involving trained health care providers, families, communities, and policymakers. Tailored interventions promoting diabetes screening, healthy lifestyles, and supportive environments, especially for women from disadvantaged backgrounds, are essential. Future research should focus on community-based strategies to overcome barriers to care and improve diabetes management, ensuring better outcomes for mothers and babies. Given the crucial role of social support, as emphasized in the literature and this study, we recommend that authorities and commissioners enhance support for community activities and organizations that foster positive social networks and peer support for pregnant women with diabetes.

## Data Availability

Data are contained within the article. Since our qualitative data resulted from confidential participant interviews, the complete transcripts of each interview cannot be released without the written consent of each participant.

## Abbreviations Used

AI: Artificial intelligence
BMI: Body mass index
GDM: Gestational diabetes mellitus
HbA1c: Glycated hemoglobin
NIMHD: National Institute on Minority Health and Health Disparities
QC: Quality control
QD: Qualitative description
SD: Standard deviations
T2DM: Type 2 diabetes mellitus
